# Training Leaders in Trauma Resuscitation: Teacher and Learner Perspectives on Ideal Methods

**DOI:** 10.5811/westjem.2021.5.51428

**Published:** 2022-02-13

**Authors:** Samantha Quon, Jeffrey Riddell, Kelsey Ford Bench, Clare Roepke, Elizabeth Burner

**Affiliations:** * Keck School of Medicine of the University of Southern California, Department of Internal Medicine, Los Angeles, California; † Keck School of Medicine of the University of Southern California, Department of Emergency Medicine, Los Angeles, California; ‡ Lewis Katz School of Medicine of Temple University, Department of Emergency Medicine, Philadelphia, Pennsylvania

## Abstract

**Introduction:**

Effective leadership improves patient care during medical and trauma resuscitations. While dedicated training programs can improve leadership in trauma resuscitation, we have a limited understanding of the optimal training methods. Our objective was to explore learners’ and teachers’ perceptions of effective methods of leadership training for trauma resuscitation.

**Methods:**

We performed a qualitative exploration of learner and teacher perceptions of leadership training methods using a modified grounded theory approach. We interviewed 28 participants, including attending physicians, residents, fellows, and nurses who regularly participated in trauma team activations. We then analyzed transcripts in an iterative manner to form codes, identify themes, and explore relationships between themes.

**Results:**

Based on interviewees’ perceptions, we identified seven methods used to train leadership in trauma resuscitation: reflection; feedback; hands-on learning; role modeling; simulation; group reflection; and didactic. We also identified three major themes in perceived best practices in training leaders in trauma resuscitation: formal vs informal curriculum; training techniques for novice vs more senior learner; and interprofessional training. Participants felt that informal training methods were the most important part of training, and that a significant part of a training program for leaders in trauma resuscitation should use informal methods. Learners who were earlier in their training preferred more supervision and guidance, while learners who were more advanced in their training preferred a greater degree of autonomy. Finally, participants believed leadership training for trauma resuscitation should be multidisciplinary and interprofessional.

**Conclusion:**

We identified several important themes for training leaders in trauma resuscitation, including using a variety of different training methods, adapting the methods used based on the learner’s level of training, and incorporating opportunities for multidisciplinary and interprofessional training. More research is needed to determine the optimal balance of informal and formal training, how to standardize and increase consistency in informal training, and the optimal way to incorporate multidisciplinary and interprofessional learning into a leadership in trauma resuscitation training program.

## INTRODUCTION

Effective leadership during trauma resuscitations requires collaboration, communication, and decisiveness.^1^–^3^ Dedicated leadership training programs can improve team performance and nonclinical skills such as identification of a team leader, role assignment, situation monitoring, mutual support, and communication.^4^–^7^ Clinical outcomes such as time to computed tomography, time to endotracheal intubation, time to operating room, total resuscitation time, percentage of task completion, and skills assessment also improve with formal team training.^4^,^7^–^10^

Targeted training of trauma leadership skills is necessary to achieve proficiency.^11^ Despite consensus on the importance of leadership training, team leadership is often a small component of broader trainings.^12^,^13^ The ATLS curriculum recommends leadership training, but does not provide specific guidelines or desired skills.^14^ Simulation training and video debriefing individually improve trauma resuscitation performance; however, their role is unclear in the milieu of other training methods.^9^,^10^ Up to 14 teaching methods have been identified in ED medical resuscitations but have not been studied simultaneouly.^15^

We do not know the extent to which informal and interprofessional education aids in acquiring trauma leadership skills, despite the significant role informal curriculum plays in resident education.^16^,^17^ Additionally, the perspectives of both learners and teachers on ideal methods of teaching communication and leadership in trauma resuscitations are not well understood. These insights into where and how training occurs can provide an important perspective to consider in the development of effective training programs. To better understand these perspectives, we explored learners’ and teachers’ perceptions of effective methods of leadership training for trauma resuscitation.

## MATERIALS AND METHODS

We performed a qualitative exploration of learner and teacher perceptions of leadership training methods using a modified grounded theory approach.

### Setting, Population and Sampling Strategy

We purposively sampled providers from different levels of training and professional backgrounds. As leaders progress in training, their perceptions of their training may change, and viewpoints from different training levels are needed for a more complete understanding of the best ways to train leaders in trauma resuscitation. Senior EM residents and trauma fellows provided the perspective of recently trained leaders, while attending physicians provided their insight from training multiple generations of trauma resuscitation leaders. Experienced ED nurses provided an important ancillary view as witnesses and participants in this training. As leadership and communication in trauma resuscitations is transdisciplinary and involves multiple specialties, we purposively sampled EM attending physicians, senior EM residents, trauma fellows, and ED nurses who regularly participate in trauma team activations (TTA) at an urban, academic teaching hospital and Level 1 trauma center. Senior EM residents and trauma-trained ED nurses were selected randomly from a list of all possible participants at our hospital. All trauma fellows participated. The EM attendings were purposively selected for those who had been at the institution for at least 10 years.

**Population Health Research Capsule**

What do we already know about this issue?

*Effective leadership improves patient care during medical and trauma resuscitations, and dedicated training programs can improve leadership in trauma resuscitation.*

What was the research question?

*We explored learners’ and teachers’ perceptions of effective methods of leadership training for trauma resuscitation.*

What was the major finding of the study?

*Perceived ideal training includes interprofessional training, informal and formal methods, and adapts to learner experience.*

How does this improve population health?

*Understanding best methods of training leaders in trauma resuscitation will contribute to better care and outcomes for critically ill trauma patients.*

Study participants were recruited through email, electronic listservs, and personal solicitation. Participants were told that the study was to understand the role of leadership in trauma resuscitations. No one who was approached to be interviewed declined. This study was approved by the local institutional review board prior to the start of data collection. Each participant was verbally consented prior to the interview.

### Protocol

The interview guide was developed with input from senior emergency and trauma physicians to explore perceptions of training strategies. The interviewer, who completed several rounds of training with mock interviews, was a graduate student at the time of data collection. Interviews were audio recorded for accuracy and professionally transcribed. Participants were given a $20 gift card to compensate for their time. We conducted 28 one-on-one, semi-structured interviews with trauma team providers between February 9–April 28, 2015 in quiet public areas and personal offices in the medical center, with no observers. Three interviews had substantial issues with audio quality and were not included in the analysis. Interview duration ranged from 12–30 minutes, with a median of 22 minutes. We conducted 56% (14/25) of interviews with female participants; 68% (17/25) participants were affiliated with the ED. This included five ED nurses, six senior EM residents, and six ED attendings. Of the participants 32% (8/25) were involved with the department of trauma surgery, which included four trauma fellows and four trauma attendings.

### Analysis

We developed a codebook and thematic structure using modified grounded theory and an iterative process.^18^ During the initial review of the interview transcripts, three coders developed an initial set of codes using three interview transcripts. Using Charmaz’s modified grounded theory methodology, themes were open coded into overarching phenomenon categories and then reanalyzed and selectively coded into categories that describe methods of leadership training in trauma resuscitation.^19^ The initial proposed codebook was then applied to four additional interview transcripts. Anonymized transcripts and codes were reviewed by a senior EM resident interviewee who added to the developing coding framework. As further participant quotations were coded and analyzed, concepts and phenomena were compared to one another to ensure the coding structure continued to accurately represent the experiences of the participants. The final codebook was further developed until theoretical saturation was reached. (See online appendix for codebook and application instructions.)

Transcripts were analyzed using Dedoose software (Sociocultural Research Consultants LLC, Manhattan Beach, CA) to organize, tally and investigate the themes identified.^19^ Two coders went through three rounds of individual coding training, and completed the embedded Dedoose inter-coder reliability test with a final inter-rater pooled kappa of 0.96, which quantified excellent level of agreement of code application between coders.^20^ Each of the remaining 18 interviews were then coded by one of the trained coders. To increase trustworthiness, we maintained an audit trail, created memos on developing codes and themes, and conducted biweekly reflection sessions with the principal investigator and coders to remain grounded in the data. The analysis was informed by the authors’ backgrounds as both students and trauma team leaders, as well as sensitizing concepts drawn from informal learning frameworks.^17^,^21^ As the transcripts were coded, themes of ideal perceptions of training methods and when to use each training methods arose and were discussed on a biweekly basis.

## RESULTS

Through analysis of a total of 175 pages of interview transcripts with 25 different participants, we identified 25 unique codes and seven training techniques (Table 1). Based on interviewees’ perceptions, we identified seven training methods used to train leadership in trauma resuscitation: reflection; feedback; hands-on learning; role modeling; simulation; group reflection; and didactic. We identified three major themes of training leaders in trauma resuscitation: formal vs informal curriculum; training techniques for novice vs more senior learners; and interprofessional training.

### Formal vs Informal Curriculum

Training methods for leadership in trauma resuscitation generally fell into informal or formal curriculum. The informal curriculum included hands-on learning, role modeling of seniors and attendings, reflection and assessment, and feedback from seniors and attendings. Informal methods were used when training was unscheduled and took place during a shift, when time or the scenario permitted. In contrast, formal techniques were used in more organized or structured training that took place outside of clinical work. These techniques included simulation, didactics. and group reflection. Group reflection included conferences such as morbidity and mortality conferences, organized group feedback, and video review. Both the formal and informal curriculum were considered valuable aspects of leadership training.

Interviewees identified informal curriculum as a crucial part of their training. Modeling by seniors and attending physicians allowed learners to develop their own style. Through role modeling, learners observed techniques they would incorporate in their future practice as well as techniques they viewed as ineffective. Feedback was an important technique because it allowed learners to receive constructive criticism and reinforcement of what they did well. Hands-on learning was viewed as the most effective method of training. Interviewees described informal training as a combination of several methods used concurrently. For example, learners first gained new leadership skills through role-modeling of seniors and improved these skills through hands-on practice. Learners continued to improve through reflection and feedback from teachers.

As one interviewee stated,

*“Top 3 things, 1 is watching my seniors* *…* *there is definitely the people who are frantic and disorganized and the forest before the trees thing, and there are people who are very calm and collected and yet effective* *…* *Attending feedback* *…* *and then mainly just doing it and messing up a ton* *…* *as you run it, you just get better.” – EM resident*

However, participants perceived drawbacks to the informal curriculum. One limitation of feedback was that it took place during a shift and was conditional on the availability of the learner and teacher to discuss feedback while simultaneously providing patient care. A negative aspect of hands-on learning was that trauma resuscitations were viewed as high-pressure environments that required speed and precision, making it difficult for learners to practice new skills. For example, as one participant explained,

*“I think that the most effective thing would probably be* *…* *when you actually run a TTA and then talking about it afterwards* *…* *it’s hard though, too, because when you’re really busy, sometimes you don’t have time to go sit down with your attending afterwards and talk for 15 minutes about how the TTA went because you have another TTA coming in” – EM resident*

Furthermore, another participant explained that

*“* *…* *a lot of it is learned by doing, I think that is hard, obviously in trauma, because it is high stakes* *…* *if you have seen it a bunch of times, a lot of time it goes a little bit more smoothly, but you have to do it for the first time at some point, and, so learn by doing definitely.” – Trauma attending*

Overall, formal training was perceived as less effective than informal techniques but was still valued. Simulation was effective because learners could scrutinize performances to identify potential weaknesses and could practice in a lower stress situation—as opposed to learning informally on a busy shift with high stakes. Participants thought didactics combined with other methods, such as simulation or hands-on training, would be most beneficial. Didactic training alone was not considered effective because interviewees also wanted more active, hands-on learning. For example, one participant described an ideal training,

*“A [refresh] on ATLS* *…* *And then, I think a lot of it would be* *…* *real hands-on training* *…* *I think a lot of it would need to be actual ‘in the moment’ events.” – EM resident*

Another participant explained:

*“For the first time, it’s kind of nerve wracking, especially if you get a trauma patient you need a chest tube and you need the rapid transfuser* *…* *yes you’ve seen it done before* *…* *but have you never done it before? It’s kind of nerve wracking if you have to do it right now ‘cause I need it right now rather than talking you through it when nothing’s going on* *…* *So that’s why simulation would be great.” – ED nurse*

Perceived drawbacks of simulation were inadequate re-creation of a pressured environment, and the time and money required to organize simulation sessions. When considering feedback, simulation training techniques, and tape review or postmortem training, interviewees preferred tape review or feedback immediately after a real-life scenario to simulation. For example, one participant stated,

*“We had a mock trauma situation and they try to make it* *…* *genuine. But, it comes off as very* *…* *fake because there’s not that sense of real urgency, which I think it’s hard to simulate.” – Trauma fellow*

### Methods for Novice vs More Senior Learners

Participants distinguished between ideal training methods for novice and more senior learners. For example, novice learners were perceived to benefit more from a low-pressure learning environment and increased support and guidance. In contrast, more senior learners required more individualized training and autonomy to prepare them for caring for patients independently.

Tape review, simulation, role modeling of seniors or attendings, didactic learning, and peer advice were identified as important training techniques for novice learners. Role modeling of seniors and attending physicians was also perceived as better for novice learners. Participants considered tape review, simulation, and didactics to be important training techniques for novice learners because it provided a way to introduce residents to leading trauma resuscitations in a lower stress environment. For example, one junior participant explained that she learned from observing others:

*Just watching them, seeing what I liked and what I didn’t like, and then* *…* *trying to model what I thought was a good leader, and just practice.” – EM resident*

Another participant found simulation to be very valuable:

*“Simulation is very important [for] practicing being decisive* *…* *That’s one of the biggest challenges for a junior resident that’s becoming a senior resident, you step into that role of just be the one to make a decision” – EM resident*

Hands-on practice and feedback were important learning techniques for more senior learners. These techniques allowed senior learners to continue to improve their existing leadership skills. Feedback after running trauma resuscitations was particularly important for more senior learners. The ideal timing of feedback changed based on the training level of the learner. For example, feedback given after a resuscitation to a senior learner allowed for more autonomy. In contrast, feedback given during a resuscitation was perceived as better for a novice learner.

One participant explained how to adjust a teaching style depending on the learner’s experience:

*“There were times when the residents were running things very smoothly, and then, maybe just give them feedback at the end of the case, try to minimally intervene … [residents] haven’t run traumas before in their PGY* [postgraduate year]* 2 year, so I’ll watch them do it, and I’ll interject comments as they are doing it” – EM attending*

### Interprofessional and Multidisciplinary Training

The third prominent theme was the benefit of interprofessional and multidisciplinary training involving trauma surgeons, emergency physicians, and nurses. This approach was perceived to improve the quality of leadership training and strengthen interdepartmental relationships. Multidisciplinary and interprofessional training were discussed in the context of simulation, role modeling, postmortem, and feedback training techniques. One strategy was for learners to observe providers from other departments, such as EM residents observing how trauma surgery fellows run codes and vice versa. Another offered example was multidisciplinary debriefs or feedback sessions with all team members after codes or trauma resuscitations to get additional perspectives on team performance. Cross-department training sessions were considered beneficial because they mimic the collaborative nature of caring for trauma patients as well as strengthening interdepartmental relationships. Nurses supported joint training sessions with nurses and doctors, such as simulations and morbidity and mortality conferences.

One participant explained how multidisciplinary training would simulate real situations:

*“You want to be as life-like as possible, and the ER doesn’t operate in isolation for a lot of these patients, to have the trauma team there running in too, and they have as much reason to benefit as we do* *…* *[Trauma is] going to have a different set of expectations” – EM attending*

Another participant stated,

*“I know they talk about* *…* *certain cases that go bad. And I think if we were invited to come to a lot more that would help overall because then if we would understand more of their perspective on* *…* *their outcomes, what their solutions are, how they would manage certain things. And then it would* *…* *help us work better as a team.” – ED nurse*

## DISCUSSION

Effective leadership in trauma resuscitation improves care; however, the best ways to train physicians to be leaders in trauma resuscitation is not fully known. From the experiences each of our participants shared, we identified several modalities currently used for training in trauma resuscitation at our institution and explored perceptions of these strategies. Participants described both informal and formal curricula that were important to training. Participants identified ideal training methods based on learner experience. Importantly, participants felt that leadership training for trauma resuscitation should be multidisciplinary and inter-professional and include ED nurses and emergency physicians, as well as trauma surgeons.

Participants identified both formal and informal curricula as important to training leaders in trauma resuscitation. Overall, informal training was perceived as more important for leadership in trauma resuscitation, consistent with prior work on the significance and benefits of informal training in residency.^16^,^22^ However, participants reported inconsistent experiences with informal training. Other studies have demonstrated this variation in learning opportunities with informal curricula,^22^–^24^ creating potential gaps in the training of learners or even exposing learners to curricula that is unapproved or may even contradict formal curricula.^22^,^23^,^25^ Our findings indicate trauma resuscitation leadership curriculum should include both formal and informal training components.

Learners’ and teachers’ perceptions of ideal training methods differed based on the learner’s level of training. Novice learners require more supervision and guidance, while more advanced learners need a greater degree of autonomy, consistent with prior work that demonstrated autonomous clinical practice is a valuable part of training,^26^–^29^ and that learners require a decreasing level of oversight as they progress.^30^–^33^ Furthermore, confronting progressively more challenging clinical scenarios based on the learner’s stage of development is itself critical for learners to progress.^32^ In our study, novice learners were perceived to benefit when teachers directly provided new information or demonstrated a skill to them (role modeling, peer advice, and didactics). More senior learners were perceived to learn best through performing a skill themselves, with less active instruction from the teacher. This highlights the importance of graduated responsibility in leadership training. Despite the barriers to progressive independence,^34^ training should initially involve techniques with an active supervisor role, with learners gradually gaining more autonomy as they gain more experience.

The importance of interprofessional and multidisciplinary training in trauma leadership was a recurrent theme in this study. Trauma resuscitations involve the combined efforts of an interprofessional team, including emergency physicians, trauma surgeons, and ED nurses.^4^,^35^,^36^ Multidisciplinary and interprofessional training improves both teamwork and clinical measurement outcomes and provides valuable experience to physicians in training.^37^,^38^ Our study complements these outcome-based studies as our participants desired increased interprofessional and multidisciplinary leadership training. These principles can also be incorporated into the assessment of the Accreditation Council for Graduate Medical Education milestones for EM: Leading interprofessional debriefing sessions after major trauma resuscitations would demonstrate level 4 competency.^39^ Emphasizing interprofessional and multidisciplinary communication and input in this competency will augment this training.

## LIMITATIONS

Our study has several limitations. It was performed at a large. urban trauma center and may not be generalizable to hospitals with a smaller volume of trauma patients or fewer resources. However, while the proportion of trauma patients seen and availability of resources such as simulation centers may vary greatly between EDs, the concepts of using both formal and informal training methods, altering the training methods used for novice and more senior learners, as well as incorporating multidisciplinary and interprofessional training would be valuable for many different training programs and should be emphasized in all settings. There is also a potential for social desirability bias, although this was minimized by making the interview transcripts anonymous to the study team and using an interviewer who was not affiliated with the training program.

Another potential limitation is that we only sought the perspectives of providers who routinely lead trauma resuscitations. At our institution only senior EM residents and trauma fellows are permitted to lead trauma resuscitations; therefore, we only interviewed PGY 4 level residents and PGY 7 level trauma fellows. (Junior EM and general surgery residents were not included in this study.) Finally, we investigated trainee perceptions of training methods and did not look at measured changes in learning, behavior, or clinical outcomes. Therefore, our participants’ narratives about the aspects of training that were most beneficial for their learning should be contextualized within the literature on the limitations of self-assessment.^40^

## CONCLUSION

In this qualitative study, we identified important methods and themes for improving the training of leaders in trauma resuscitation based on the perspectives of both learners and teachers. We recommend that training programs in leadership for trauma resuscitations consist of a variety of training methods, adapt based on the learner’s level of training, and incorporate both multidisciplinary and interprofessional training. Future studies should include outcomes-based research to determine whether these strategies result in improved leadership skills and clinical performance*,* as well as longitudinal studies with recent graduates to explore changes in perceptions of training as they transition from learner to teacher.

## Supplementary Information



## Figures and Tables

**Table 1:** Summary of identified training methods and relationship to major themes.

Training method	Definition	Informal vs formal	Best for novice vs more senior learners	Quote
Reflection	The learner analyzes his/her own performance	Informal training	All levels	*…if things don’t go well or we look back and [I] think to myself … that took a long time in the ER, was there something that I could have done sooner to get the patient either to the OR or ICU … why did the work-up take that long? – trauma attending*
Feedback	The teacher gives the learner feedback regarding their performance in real practice in an informal setting (i.e., during a shift)	Informal training	More senior training	*There is no formal system, usually the attendings will just pull you aside and say, hey, what do you think went well, [or] if something was clearly wrong … there is no formal system. – EM attending* *Absolutely … if something isn’t perfect then … we’ll address it afterwards … debriefing is always important, especially if something goes wrong. - trauma attending*
Hands-on learning	Learners get training through real-time, hands-on/direct experience in treating real patients in trauma scenarios (rather than in practice scenarios or simulation)	Informal training	More senior training	*There’s no preparation enough; it’s the experience of it where you start to learn … you really get that on the job training so to speak. – EM attending* *Ultimately, I would say that you can’t learn it just by observing, you got to live it. – EM attending*
Role modeling	Learners observe peers, seniors, and attendings in their real practice, good and bad examples	Informal training	Novice training	*My previous leaders, basically, just watching them, seeing what I liked and what I didn’t like, and then … trying to model what I thought was a good leader, and just practice. – EM resident*
Simulation	Leadership training through practice scenarios, e.g., sim lab, Advanced Trauma Life Support, mock scenarios.	Formal training	Novice training	*I think … simulation is very important… [for] walking through-- what would you do in this situation? Just practicing, being in that role of being decisive. – EM attending*
Group reflection	Leadership training through an organized or formal event, or feedback given in a group format, e.g., morbidity and mortality conferences, grand grounds, or tape review.	Formal training	All levels	*It would be … more of a situation where you … videotape a TTA as it’s happening and then, postmortem, going back and reviewing it with … all the people involved-- I think that would be helpful. – trauma fellow* *One of the things that I’ve wanted to do is seeing if we can get cameras in there … Because people don’t know what they don’t do or what they do do, until they see it. – trauma attending*
Didactic	Learners gain leadership skills through formal lecture-based learning	Formal training	Novice training	*That’s tough to do in terms of just … sitting down in a classroom in a lecture type thing, it definitely needs to be more of an active participation type thing. – trauma fellow*

*TTA*, trauma team activation.
