# Supplementary material for: Training Leaders in Trauma Resuscitation: Teacher and Learner Perspectives on Ideal Methods
Source: West J Emerg Med. 2022 Feb 13;23(2):192–9. doi: 10.5811/westjem.2021.5.51428 (PMC8967462; doi:10.5811/westjem.2021.5.51428)
Supplement: Supplementary file 1 [file wjem-23-192-s001.docx]

**Appendix**

**Training Leaders in Trauma Resuscitation: Interview Guide**

1. How long have you been in your current position? How does it relate to trauma care?
2. Describe any other roles you’ve had in caring for trauma patients.
3. Describe what makes a successful Trauma Team Activation (TTA).
4. Describe what makes someone a good leader during a trauma resuscitation.
   1. Do the same things make a good leader in other areas of patient care?
   2. What about in teaching situations?
5. Do you consider yourself to be a leader? Why? In what context?
6. Describe any training or education you received on effective leadership. How did you develop your leadership skills?
7. Describe situations in which your leadership style changes.
   1. Does the style change if residents are present? Based on the clinical scenario? Severity of the trauma?
8. Describe a challenging trauma situation you have had.
9. How does team communication effect team performance in trauma?
10. If LAC+USC were to institute a leadership training program, what would that look like?

**Codebook: Codes, definitions and coder guidance**

| **CODE** | **DEFINITION/GUIDE FOR USAGE/COMMENTARY** |
| --- | --- |
| “Content” | Clear delegation of leadership tasks, persona, clear communication, managing expectations, managing tone; skills and topics that should be taught in a course on trauma resuscitation leadership training |
| “Emulation” | Learners observing teachers in their real practice to develop leadership style, both observation of perceived good and bad examples |
| “Simulation” | Simulation lab, ATLS, good and bad aspects of simulation. Good aspects includeless stress, exposure to flaws, repetition in simulation increases skills, active learning. Bad aspects include itinvolves extra time, involves money expenditure) |
| “Feedback “ | Giving feedback re: real practice, receiving feedback, either in public or private, **one on one or if unclear if individual** |
| “Postmortem” | Delayed or immediate group feedback, outside views with group (delayed). Examples includemorbidity and mortality conference, tape review. **GROUP (not individual) event~~;~~** an organized event (not spontaneous) |
| “Learning by doing” | Get experience by doing the real work directly with patients in real time, rather than in practice/simulation |
| “Didactics” | Classroom session that teaches leadership for learners, formal training. Examples includelectures, ATLS |
| “Repetition” | Practice makes perfect, practice improves time/success in the real situation, practice improves comfort |
| “Self Reflection/Assessment” | Checking self reflection with trainer, verbalizing self-reflection, trainer encouraging self-reflection |
| “Peer:peer advice” | Learning from departing seniors, (verbal NOT emulating) |
|  |  |
| “MultiSpecialty” | **CO-CODE ONLY** with Method, review with members of different specialties or nurses/cross specialty |
| “Good” | **CO-CODE ONLY** with Method, specific Positive aspects; better than other methods |
| “Bad” | **CO-CODE ONLY** with Method, specific Bad aspects; worse than other methods |
| “Teaching Strategy” | **CO-CODE ONLY** with method; strategies used to teach residents/student to be leaders |
|  |  |
| “Lack of systematic training” | Any reference to lack of formal training, including comparison to other markets |
| “Gender differences” | Difference in what is told to male/females during training |
| “Who gets trained” | When do people get trained, is training mandatory, training as voluntary, what year, what level, who does the training |
| “Training the educator” | Teaching how to be an educator, knowing how to teach as a valuable tool |
| “Doing not same as leading” | Differentiating skills vs leadership, people with good skills not inherently good teachers |
| “Stress” | Communicating in a stressful environment, stress & clinical pace influencing training |
| “More than 1 ideal style” | Multiple styles of leadership in trauma/practice styles |
| “Teacher/Learner interaction” | Approving decisions/seeking approval, learner asserting authority, reinforcing learner authority, verbal & non-verbal affirmation, physical / verbal presence, directions from teacher during real life care |
| “Graduated Responsibility” | Giving increasing responsibilities to learner as they progress |
